# SUMOFLUX: A Generalized Method for Targeted ^13^C Metabolic Flux Ratio Analysis

**DOI:** 10.1371/journal.pcbi.1005109

**Published:** 2016-09-14

**Authors:** Maria Kogadeeva, Nicola Zamboni

**Affiliations:** 1 Institute of Molecular Systems Biology, ETH Zürich, Zürich, Switzerland; 2 Life Science Zürich Graduate School, Zürich, Switzerland; The Pennsylvania State University, UNITED STATES

## Abstract

Metabolic fluxes are a cornerstone of cellular physiology that emerge from a complex interplay of enzymes, carriers, and nutrients. The experimental assessment of *in vivo* intracellular fluxes using stable isotopic tracers is essential if we are to understand metabolic function and regulation. Flux estimation based on ^13^C or ^2^H labeling relies on complex simulation and iterative fitting; processes that necessitate a level of expertise that ordinarily preclude the non-expert user. To overcome this, we have developed SUMOFLUX, a methodology that is broadly applicable to the targeted analysis of ^13^C-metabolic fluxes. By combining surrogate modeling and machine learning, we trained a predictor to specialize in estimating flux ratios from measurable ^13^C-data. SUMOFLUX targets specific flux features individually, which makes it fast, user-friendly, applicable to experimental design and robust in terms of experimental noise and exchange flux magnitude. Collectively, we predict that SUMOFLUX's properties realistically pave the way to high-throughput flux analyses.

## Author Summary

Living cells adapt to ever-changing environments by regulating metabolic fluxes, the rates of nutrient flow through the metabolic network, to produce metabolites that are currently in demand. ^13^C-labeling techniques coupled with metabolic flux analyses are widely used to estimate metabolic fluxes and provide insights into cellular physiology and adaptation relevant in biological, biomedical and biotechnological applications. However, the existing methods are either computationally costly, or applicable to a limited amount of biological systems. Here, we combined **su**rrogate **mo**deling with machine learning to present SUMOFLUX, a generalized method for ^13^C flux ratio analysis. We validated our method by resolving canonical flux ratios in eight *Escherichia coli* mutants with known metabolic phenotypes and estimated a novel flux ratio for this dataset. We demonstrated scalability of SUMOFLUX and its application for experimental design by applying it to a cohort of 121 *Bacillus subtilis* mutants. SUMOFLUX, alone or in combination with global flux analysis methods, can be applied to resolve flux ratios in virtually any biological setup, and paves the way to high-throughput flux profiling.

This is a *PLOS Computational Biology* Methods paper.

## Introduction

Metabolic fluxes describe the *in vivo* flow of organic matter through the biochemical reaction network, as defined by enzymes and transporters. An improved knowledge of metabolic fluxes is crucial if we are to understand how cells utilize nutrients, and how they regulate metabolism in the face of dynamic environmental conditions, or in stressed pathologic states [1–4]. Metabolic fluxes, as an emergent property of cellular systems, are prohibitively hard to predict using proteomics or metabolomics data, and are not, per-se, measurable. Hence, the task of assessing metabolic fluxes indirectly represents something of an analytic and mathematic tour-de-force.

The most informative approach to estimate metabolic fluxes involves stable isotope labeling. Cells grown in the presence of ^13^C-enriched substrates incorporate heavy isotopes throughout their metabolic networks according to carbon fluxes and produce characteristic ^13^C patterns in metabolites and products. Some of these can be measured by mass spectrometry or nuclear magnetic resonance and can ultimately be used to deduce fluxes using two basic approaches. The first is global isotopomer balancing, which seeks to estimate all metabolic fluxes by iterative fitting [5–10]. The power of this approach is that it integrates all available data simultaneously in order to estimate metabolic fluxes across the entire system. The downside is that this approach is ill suited for high-throughput analyses as it necessitates quantification of all uptake and production rates, and analyzes each sample individually. In addition, the fitting procedure is mathematically cumbersome, and for complex or poorly calculable problems, can require extensive computation time. Finally, troubleshooting heavily relies on expert knowledge [8].

The alternative approach is to use flux ratio analysis, which focuses on the resolution of local fluxes, centered on metabolic nodes of particular interest [11–16]. For this purpose, flux ratio analysis adopts a targeted strategy in which relative (fractional) information on contributions from alternative pathways are calculated from a small subset of ^13^C-data using predefined analytic formulas. The advantage of this approach is that it is mathematically simple, rapid, well suited for large scale analyses [16], and easily used by the non-expert user. However, this process suffers from the time-consuming procedure of deriving analytic formulas for each flux ratio of interest. These formulas, manually derived for each metabolic network, tracer, and environment, generally incorporate a mix of human intuition together with tacit assumptions regarding flux. Over the past 20 years, only a dozen have been derived to describe the central metabolism of microbes growing on single carbon sources. In practice, most experimental conditions cannot be addressed due to the lack of validated flux ratio predictors. In response to these limitations, automated tools have been developed to estimate flux ratios [17,18], although, thus far, these have been limited to linear cases and consequently have failed to find any broader application.

Here we present SUMOFLUX, a conceptually novel method to analyze, in a targeted fashion, flux ratios based on ^13^C-data. Our workflow circumvents concerns over the relevance and limitations of flux analyses by exploiting machine learning. A machine learning predictor is trained using *in silico* ^13^C-data, generated by surrogate modeling. The combination of surrogate modeling and machine learning permits the rapid estimation of flux ratios for virtually any metabolic network, label configuration, or available measurement. We now illustrate the proposed workflow for both canonical and novel flux ratios for central carbon metabolism. The speed and generality provided by machine learning makes SUMOFLUX particularly useful for optimizing experimental design, selecting metabolites to be measured, and merging data from several experiments. Moreover, we believe that the SUMOFLUX workflow provides a real prospect of high-throughput flux analyses.

## Results

### Surrogate modeling and the workflow of ^13^C-flux ratio analysis

In ^13^C-metabolic flux ratio analysis, the goal is to estimate a flux ratio of interest. Typically, this is a number that indicates the relative fraction of a specific metabolite flowing through a chosen reaction or pathway. Flux ratios are estimated based on a stoichiometric model, knowledge of the ^13^C-configuration of all of the relevant substrates, and the labeling patterns of metabolites as measured by mass spectrometry (or nuclear magnetic resonance). We formulated the derivation of flux ratio estimates from ^13^C data as a nonlinear regression task to be solved using machine learning. By definition, the flux ratio of interest is the dependent variable that we aim to predict; measured ^13^C isotope labeling patterns of intracellular metabolites are the independent variables, or the input features for the algorithm. A random forest predictor [19] is then trained to build a functional relationship between the ^13^C data and flux ratios using a training dataset. To build a generalized predictor, the training dataset should comprise hundreds, if not thousands of representative examples for which a flux ratio and ^13^C data are available. Unfortunately, such a dataset is not accessible experimentally. First, because flux estimates are not amenable to direct measurement. Second, in the majority of cases it is impossible to select, or to construct, a cohort of cells with a phenotypic diversity that adequately represents the wide variety of fluxes and flux ratios that might exist. To overcome this fundamental problem, we have used **su**rrogate **mo**deling (hence the term SUMOFLUX). We built, *in silico*, a synthetic cohort of representative data points. Each data point is defined by a complete set of fluxes that fulfill the stoichiometric constraints of the metabolic network. This allows us to calculate a ratio (or any other derivative value) for the fluxes of interest, and to simulate the ^13^C-labeling patterns of each metabolite, which is made possible because each flux distribution leads to a unique intracellular labeling pattern [20]. It is therefore possible to construct an *in silico* dataset comprising thousands of data points, with flux ratios spanning the feasible range, and corresponding metabolite ^13^C-labeling patterns. The synthetic *in silico* dataset is used to train, cross-validate, and then test the flux ratio predictor.

**Fig 1:**
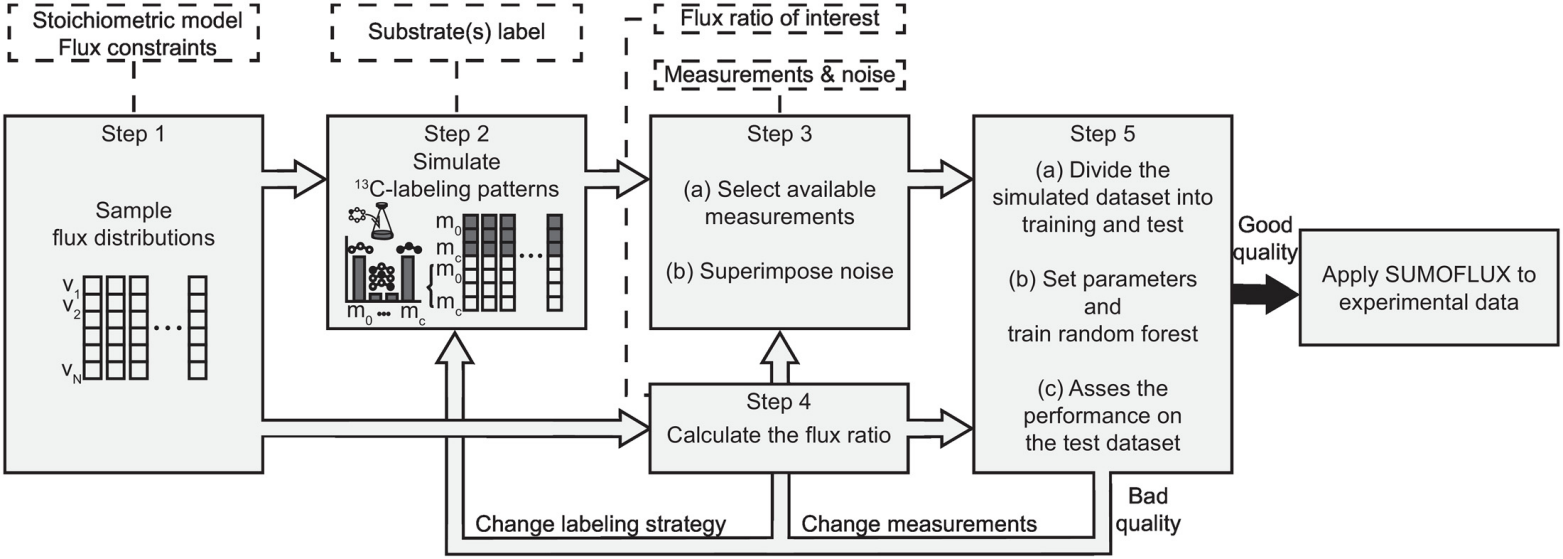
SUMOFLUX workflow for targeted flux ratio analysis. Input data are depicted in the dashed-line rectangles.

The full SUMOFLUX workflow for flux ratio estimation consists of five steps (Fig 1). First, a reference dataset of several thousand flux-maps is sampled from that space of flux-maps that fulfills certain stoichiometric constraints (mass balances) of the metabolic network. Extracellular flux constrains can be further refined by the availability of substrates and a working knowledge of the major secreted products. Second, the ^13^C-labeling patterns of the metabolites included in the network are simulated independently for each reference data point. Label propagation is simulated using the existing algorithms [9], given the ^13^C-label of the substrate(s), and the map of the atom transition within the network. At this point, the simulated ^13^C data, does not, as yet, reflect actual measurements. Third, to capture measurement data, we select only those ^13^C features that are analytically accessible, and then superimpose noise values corresponding to those measured. Fourth, flux ratios of interest are calculated for all of the data points within the reference dataset, as the dependent variable in regression analyses. Fifth, we divide the reference dataset into independent training and test subsets, using the former to train a random forest with which to predict the calculated flux ratios from simulated ^13^C data. We then assess the predictor’s performance on the test dataset by calculating the mean absolute error of the predictions made. If the performance is insufficient (e.g. mean absolute error MAE > 0.05), we iteratively optimize our experimental strategy by changing the substrate label, or available measurements, then repeat the training. If the performance is judged to be satisfactory, we finally use the predictor to estimate flux ratios using real experimental data. To provide prediction intervals, we use quantile regression forests, which give a non-parametric and accurate estimates of conditional quantiles based on the full conditional distribution of the dependent variable [21].

The most time consuming aspect of the workflow is the simulation of ^13^C data in the reference dataset, which scales according to the number of samples and carbon atoms in the metabolites. For the model of central carbon metabolism, with 39 reactions and 21 measured metabolites and fragments (S1 Fig, S1 and S2 Tables), 0.2 seconds are needed to simulate the labeling patterns for a single data point. Using a parallelization technique, this process can be accelerated to simulate the several thousand data points necessary for training and testing within a few minutes. Without parallelization, the simulation procedure for 20,000 data points takes ~1.5 hours, whereas the flux sampling and random forest training steps require less than a minute.

Overall, the SUMOFLUX workflow requires information on the stoichiometry of the metabolic network, and the carbon atom arrangement for all of the metabolites within the network. The choice of ^13^C-tracer depends on the flux ratio of interest [8,22], but in practice is primarily constrained by commercial availability and costs. Hence, it is quite common to test flux calculability using multiple configurations of tracers [23–25], which can be easily accomplished using the SUMOFLUX workflow due to its rapid computational time. In the following sections, we demonstrate the performance, generality, and scalability of SUMOFLUX, as well as its versatility in terms of feature selection and experimental design.

### Analyses of flux ratios for central carbon metabolism

We chose to demonstrate SUMOFLUX by deriving estimates of the flux ratios for central carbon metabolism using the model organism, *Escherichia coli*. Its metabolic network includes the highly conserved pathways of glycolysis, the tricarboxylic acid (TCA) cycle, and the pentose phosphate pathway (PPP). Furthermore, it includes alternative pathways such as the Entner-Doudoroff pathway and the glyoxylate shunt that conveys additional metabolic elements that might complicate flux estimates. As a reference, we considered the study of glucose metabolism in *E*. *coli* as described by flux ratio analyses using manually derived analytic equations [15]. We used our method to estimate five key flux ratios based on the labeling patterns measured by gas chromatography mass spectrometry (GC/MS) of proteinogenic amino acids upon silylation. We then sampled a reference dataset of 60,000 flux distributions using the *E*. *coli* central carbon metabolism network (S1 Fig, S1 Table), and simulated the labeling patterns of 21 intracellular metabolites and their fragments (S2 Table), assuming growth on either 100% [1-^13^C] glucose, or a mix of 20% [U-^13^C] glucose and 80% naturally labeled glucose.

Several parameters had to be defined prior to predictor training. The performance of the random forest depends on the number of decision trees in the forest (*ntree*), and the number of input features used at each tree node (*mtry*). To choose these parameters we used five-fold cross-validation on the training dataset. We tested 16 combinations of *ntree* and *mtry* values for the five *E*. *coli* flux ratios. The combination of 100 prediction trees (*ntree*) with 20 *mtry* features delivered a good balance between predictor accuracy and computation time (S2a Fig). These two parameters were then applied throughout the study. The number of simulated points used for training also influences predictor accuracy and computation time. Our tests demonstrated that ~10,000 simulated points were generally adequate in terms of generating a sufficiently accurate estimate of the key flux ratios in the *E*. *coli* dataset; thereafter, any further increase in the number of data points provided no tangible improvement in accuracy (S2b Fig). We took these results into account when extracting the training sets for the predictors (see Materials and Methods for details).

**Fig 2:**
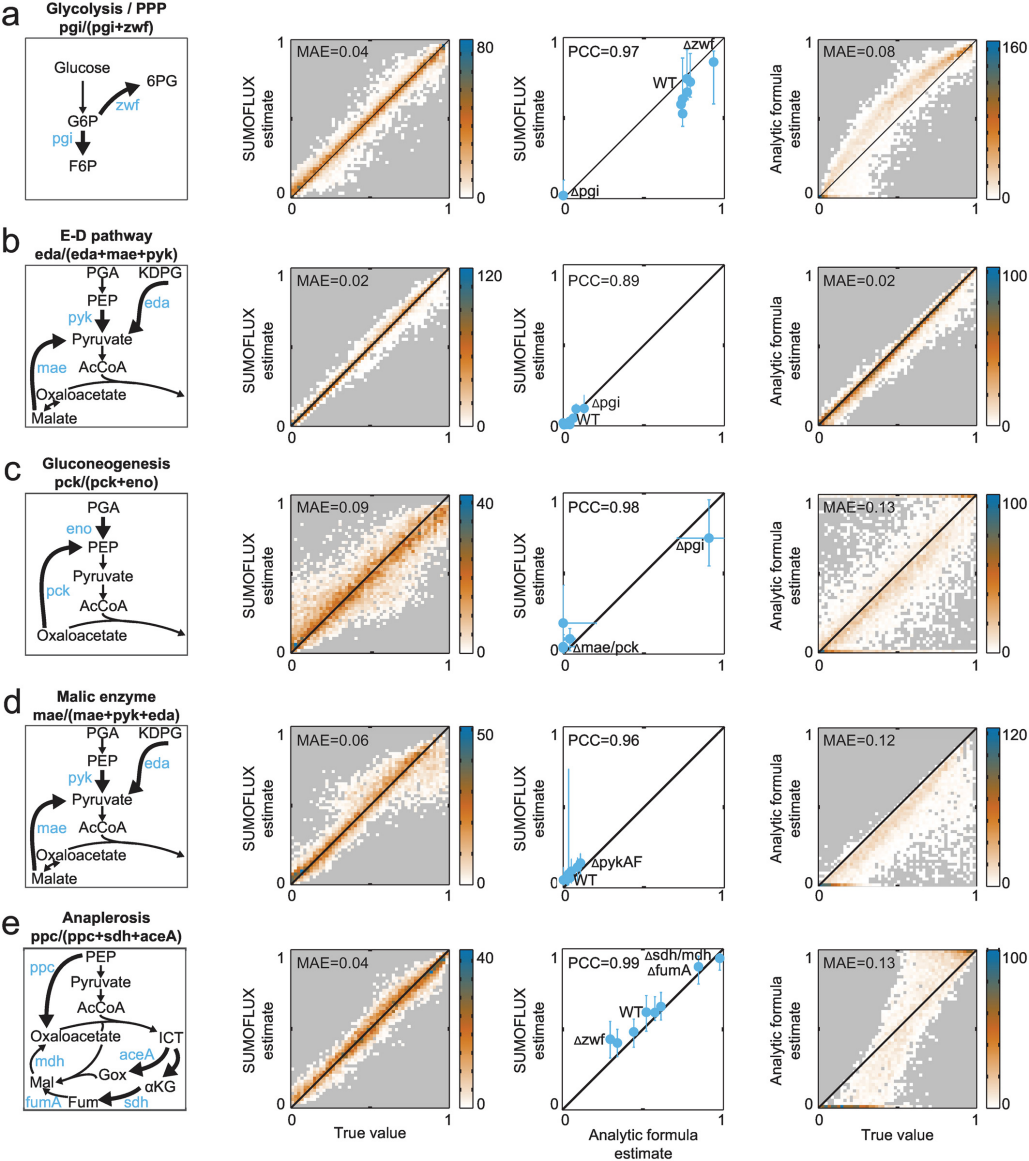
Comparison of SUMOFLUX and analytic formula estimates for flux ratios in *E*. *coli* central carbon metabolism. From left to right: a schematic representation of the flux ratio; density plot representing SUMOFLUX estimates versus the true flux ratios for *in silico* data; comparison of the SUMOFLUX and analytic formula estimates for the experimental data; density plot representing analytic formula estimates versus the true flux ratios for *in silico* data. Vertical error bars in the third panel represent [10–90%] SUMOFLUX prediction quantiles, horizontal error bars represent standard deviation provided with the analytic formula estimate. (a) Glycolysis versus PPP. (b) Pyruvate fraction from the E-D pathway. (c) PEP fraction from gluconeogenesis. (d) Pyruvate fraction from the malic enzyme flux. (e) Oxaloacetate fraction from anaplerosis from PEP. Ratios (a)-(c) were estimated from [1-^13^C] glucose experiment, ratios (d) and (e) were estimated from 20% [U-^13^C] and 80% naturally labeled glucose experiment. 6PG– 6-phosho-D-gluconate; αKG– α-ketoglutarate; AcCoA—acetyl-CoA; E-D—Entner-Doudoroff pathway; F6P –fructose-6-phosphate; Fum—fumarate; G6P –glucose-6-phosphate; Gox—glyoxylate; ICT—isocitrate; KDPG—2-Keto-3-deoxy-6-phosphogluconate; MAE—mean absolute error; Mal—malate; PCC—Pearson correlation coefficient; PEP—phosphoenolpyruvate; PGA—phosphoglycerate; PPP—pentose phosphate pathway.

We trained predictors for the five *E*. *coli* flux ratios on the simulated training dataset and then assessed their performance on an independent simulated test dataset. In all cases, the mean absolute error was < 0.1 (Fig 2, second column). For comparison, we also applied the analytic formulas manually derived for the *E*. *coli* study [15] (S3 Table) to the same simulated test dataset. For all tested flux ratios, SUMOFLUX outperformed the analytic formulas in terms of mean absolute error on the test dataset (Fig 2, fourth column). This possibly reflects the fact that the flux estimates for the test dataset were obtained through sampling of the entire solution space, and do not comply with some of the implicit simplifications and assumptions for the network, fluxes, and reaction reversibility, that are generally used to derive the analytic formulas [15]. For example, in calculating the fraction of oxaloacetate from phosphoenolpyruvate, the flux through the glyoxylate shunt was assumed to be zero, whereas in the test set it possessed a wide range of values (Fig 2e). Furthermore, the analytic formula for estimating the malic enzyme flux ratio provides only a lower bound value (Fig 2d). We also compared flux estimates generated by the two approaches using the real experimental ^13^C data from this study (Fig 2, third column, and S4 Table); both produced concordant estimates (Pearson correlation coefficient, PCC > 0.89 for all ratios).

To further demonstrate the scalability and generality of SUMOFLUX, we applied the same approach and parameters to estimate four flux ratios using the GC-MS data for amino acids collected for 121 *Bacillus subtilis* transcription factor mutants grown on a mix of 80% [1-^13^C] glucose and 20% [U-^13^C] glucose [16]. Again, the random forest predictor outperformed the analytic formulas for the *in silico* test dataset (S3 Fig). For three flux ratios, the two approaches provided consistent estimates for the experimental data (PCC > 0.65). However, the malic enzyme ratio could not be resolved with sufficient precision using either method. Presumably, the mix of tracers chosen was poorly suited to this task.

In order to highlight the scope of SUMOFLUX applicability in context of global ^13^C flux analysis methods, we compared it with the classical ^13^C-metabolic flux analysis by global isotopomer balancing (^13^C-MFA) approach, which seeks for a global flux solution that provides the best fit to the experimental data—measured metabolite labeling patterns and physiological parameters. We applied ^13^C-MFA to the data for the same eight *E*. *coli* strains and added glucose uptake rates [15] as an additional input. With INCA [9], we calculated the best flux fit and flux confidence intervals using parameter continuation procedure (S5 Table). SUMOFLUX and ^13^C-MFA differ in the demand of input information and produce different outcomes (net fluxes vs. flux ratios). To compare, we calculated flux ratios from the net fluxes estimated by ^13^C-MFA and directly compared to SUMOFLUX results. Confidence intervals on flux ratios for ^13^C-MFA were obtained by repeating the optimization procedure 1000 times for each strain. Because it employs less input data, SUMOFLUX is expected to be worse than ^13^C-MFA. In general, however, the flux ratio estimates obtained with the two methods were in good agreement (PCC>0.83 for all ratios calculated for the best fit to either [1-^13^C] data, [U-^13^C] data, or combined dataset, S4 Fig). Surprisingly, in several cases the confidence intervals of ^13^C-MFA flux ratio estimates were much larger than the prediction quantiles of SUMOFLUX and the accuracy of flux ratio estimates depended on the experimental dataset used for the fitting, perhaps pointing to the presence of inconsistent or overly noisy data that decrease the precision of ^13^C-MFA estimates. This example illustrates the complementarity of the two approaches. ^13^C-MFA provides global flux solutions, but in some cases the targeted approach performs better in resolving local fluxes.

Collectively, the *E*. *coli* and *B*. *subtilis* results demonstrate that SUMOFLUX is broadly applicable to real experimental data with an accuracy that is comparable, if not better, than that of manually derived formulas. Even though there is no guarantee that a specific flux ratio can be accurately estimated for a given metabolic network, tracer, or experimental data, SUMOFLUX does allow for rapid verification and ad-hoc experimental design. Beyond the speed and ease with which predictors can be generated for calculating metabolic flux ratios from ^13^C data, SUMOFLUX offers the additional benefits of robust prediction, the option to vary and optimize experimental design, and the estimation of novel ratios that we explore in the next sections.

### SUMOFLUX is robust in terms of experimental noise and reversible reactions

**Fig 3:**
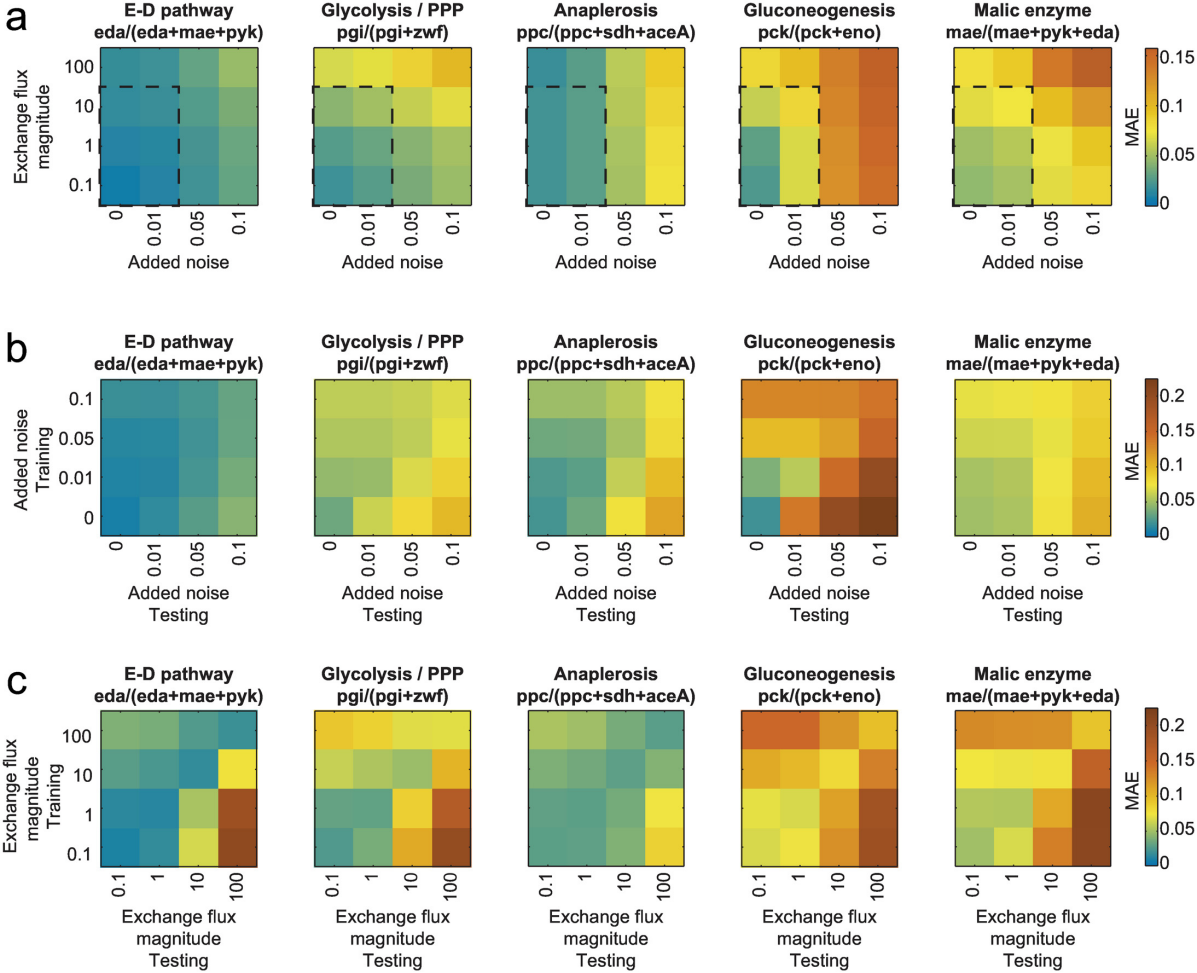
SUMOFLUX is robust in terms of experimental noise and exchange flux magnitude. (a) Mean absolute errors on the testing dataset of five flux ratio predictors applied to *in silico* data with different amount of measurement noise and exchange flux magnitude. The dashed rectangle indicates the normal range of noise (0.01) and exchange flux magnitude (10 times the net flux). (b) Mean absolute errors on the testing datasets with different noise levels of five flux ratio predictors trained on datasets with different amount of measurement noise. The exchange flux magnitude was set to 1 for all datasets. (c) Mean absolute errors on the testing dataset with different exchange flux magnitudes of five flux ratio predictors trained on datasets with different values of exchange flux magnitude. The noise level was set to 0.01 for all datasets. E-D—Entner-Doudoroff pathway, MAE—mean absolute error; PPP—pentose phosphate pathway.

Excessive measurement noise and underestimates of exchange flux of bidirectional reactions are two frequent causes of inaccurate flux estimates. We set out to assess their influence on SUMOFLUX by performing an *in silico* experiment using *E*. *coli*, varying the values of the superimposed measurement noise by up to 0.10, i.e. 10-fold higher than that routinely obtained with careful peak integration. We also used exchange flux values of up to 100-fold that of the net flux value, i.e. a model approximation of full equilibration of the reactants. To exclude potential prediction accuracy differences arising from different training and test datasets, we used one set of flux vectors, divided into training and test subsets. Addition of four different noise levels and variation of four exchange flux magnitude values resulted in 16 datasets, which differ only in these two parameters. We again trained the predictors for each of the datasets using the training subset and calculated the mean absolute error (MAE) on an independent test subset. As a rule of thumb, we consider a ratio to be accurately predictable if the MAE < 0.05. This criterion was met by the Entner-Doudoroff, glycolysis/PPP, and anaplerosis ratio predictors, within the normal ranges for noise (~ 0.01) and exchange flux (~10 times the net flux value) (Fig 3a). The other two tested predictors were less precise, and are better suited to the analysis of substantial flux changes. Alternatively, different tracers and measurement techniques could be tested, as outlined below, to achieve more accurate analyses. We also performed robustness analyses of the analytic equations, and found that only the formula for the Entner-Doudoroff pathway was sufficiently robust in terms of noise and flux exchange that were within the normal ranges (S5 Fig). The remaining four formulas were either extremely sensitive to noise (gluconeogenesis ratio), or were poorly suited to the entire range of flux maps.

Another important aspect that can be assessed with this type of analysis is to what extent erroneous assumptions in the training dataset affect the accuracy of the flux estimates in the test dataset. We used the simulated data described above to test the effects of noise and exchange flux magnitude values separately. First, we fixed the exchange flux magnitude to 1, and calculated the accuracy of the flux ratio predictors trained and tested on 16 combinations of train and test subsets with the four independently added noise levels. Notably, we observed that for all ratios underestimating the level of noise in the data was detrimental for the flux ratio prediction accuracies. On the contrary, overestimating the noise in the training data resulted in better flux estimates in the less noisy test datasets compared to the test data with the same levels of noise as in training (Fig 3b). In practice, it is always desirable to only superimpose a realistic level of noise to *in silico* data, as the addition of noise inevitably decreases the prediction accuracy. However, it is advisable to adopt a conservative over-estimate noise to avoid overfitting.

Adequate magnitude of exchange fluxes appears to be even more important for predictor accuracy. We observed that both under- and over-estimated exchange flux magnitude values resulted in lower accuracy compared to the accuracy on the test dataset corresponding to the training (Fig 3c, diagonal values). Remarkably, in these simulated datasets we set one exchange value as upper bound for all reversible fluxes in the model, which presumably has a greater effect on prediction accuracy than an exchange flux magnitude of a single reaction. In biological systems, we expect large differences in the reversible flux magnitudes of different enzymes, and it is beneficial to include this information in the model, when available.

One way to control whether the simulated data used for training and testing adequately represents the experimental data, is to compare the distributions of inter-quantile ranges of the flux ratio estimates for the experimental data to the ones of the test data. In our examples, the distributions of the inter-quantile ranges of the flux estimates for under-estimated noise or inappropriately estimated exchange flux estimates were significantly different (p<0.01, Wilcoxon-Mann-Whitney test, S6 Fig). In practice, it is advisable to compare the inter-quantile range distributions of the flux ratio predictions for *in silico* and experimental data, although statistical tests should be used with caution due to very different sample sizes.

In summary, SUMOFLUX provides flux ratio predictors that are generally robust to noise and exchange fluxes, both of which are major confounding factors in labeling experiments. This robustness is dependent on flux ratio, labeling strategy, and the available measurements used for prediction, and can easily be assessed, if required, in each particular case.

### Estimation of a novel flux ratio for the glyoxylate shunt

**Fig 4:**
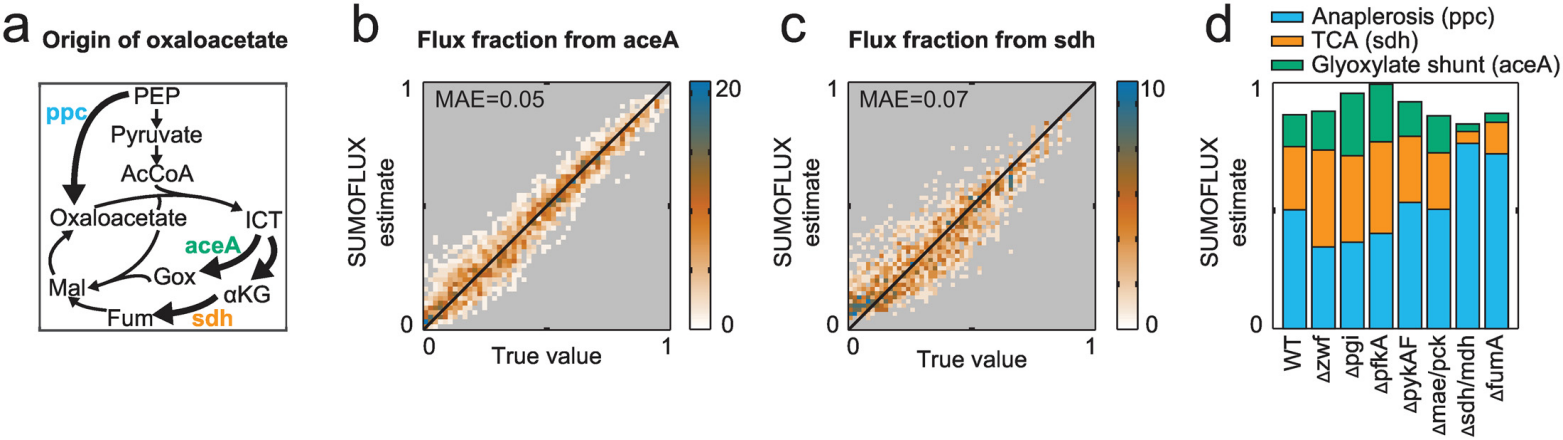
SUMOFLUX resolves a novel flux ratio in central carbon metabolism of *E*. *coli*. (a) A schematic representation of the glyoxylate shunt, TCA cycle and anaplerosis from PEP flux fractions. (b) Density plot representing SUMOFLUX estimates for the flux fraction from glyoxylate shunt versus the true flux ratios for *in silico* data. (c) Density plot representing SUMOFLUX estimates for the flux fraction from the TCA cycle versus the true flux ratios for *in silico* data. Both ratios were resolved for experiment with 20% [U-^13^C] and 80% naturally labeled glucose. (d) Predictions for the three flux fractions for the experimental data. αKG– α-ketoglutarate; AcCoA—acetyl-CoA; Fum—fumarate; Gox—glyoxylate; ICT—isocitrate; MAE—mean absolute error; Mal—malate; PEP—phosphoenolpyruvate; TCA—tricarboxylic acid cycle.

The glyoxylate shunt plays an essential role in bacterial adaptation to alternative carbon sources, such as acetate and fatty acids, as it replenishes the TCA cycle with C_2_ carbon fragments. Hence, this pathway has an important anaplerotic function besides phosphoenolpyruvate carboxylase. No analytic formulas were developed to resolve the relative contribution of the glyoxylate shunt due to the complexity of carbon rearrangement at this branch point, the additional complication introduced by multiple cycling in the TCA cycle, and the similarity of the labeling patterns of the relevant metabolites. Here, we opted to tentatively resolve this pathway using SUMOFLUX, and the ^13^C-data available from GC-MS analyses of protein-bound amino acids in *E*. *coli* [15]. Using the same simulated dataset described above, we trained two more predictors to estimate flux contributions to the formation of oxaloacetate, one derived from the glyoxylate shunt, and the other, from the TCA cycle (Fig 4a). The accuracy of the predictors achieved for the *in silico* test dataset was acceptable (MAE < 0.07) (Fig 4b and 4c). Collectively, these two novel ratio predictors, and the one previously trained to estimate the anaplerotic reaction from phosphoenolpyruvate to oxaloacetate (Fig 2e), allowed us to comprehensively assess the metabolic source of oxaloacetate. The prediction intervals of the estimates for the experimental data were in the range of 10% due to the difficulty of precisely resolving the glyoxylate shunt based on the available data. Nevertheless, the estimated values reflect those expected from the literature. Specifically, the differences between strains were consistent with their genotype (Fig 4d, S7 Fig, S6 Table). The estimated glyoxylate shunt contribution for wild type bacteria was 16 ± 10%, with the highest glyoxylate shunt ratio (32 ± 15%) estimated for the Δpgi mutant, which is consistent with other studies [26,27]. In contrast, both the double Δmdh Δsdh mutant and the ΔfumA mutant, in which the pathway from succinate to malate is disrupted, had an almost zero glyoxylate shunt and TCA cycle activity, with the major contribution to the oxaloacetate pool being the flux derived from phosphoenolpyruvate. The Δzwf mutant, with a compromised oxidative pentose phosphate pathway, exhibited the highest fraction for the TCA cycle flux (49 ± 18%), which reflects a compensatory response to ensure NADPH equilibrium via isocitrate dehydrogenase [26]. The glyoxylate shunt example shows how novel quantitative flux predictors can be rapidly generated using our approach.

### Experimental design

In the context of metabolic analyses, *a priori* experimental design aims at identifying the best settings from simulated data with which to accurately estimate the fluxes of interest. In global isotopomer balancing and fitting, numerical simulations have been frequently used to optimize tracer selection for one specific flux state, e.g. that of an unperturbed wild-type strain [23–25]. In targeted flux ratio analysis with manually derived analytic equations, simulation-assisted experimental design is not possible, as each equation is formulated for a specific experimental condition chosen by the researcher, and no simulation procedure is employed to assess its accuracy. In contrast, the speed and simplicity of SUMOFLUX facilitates the rapid testing of altered metabolic models, tracer choices, or data sets for the derivation of a flux ratio of interest. This enables us to systematically, yet rapidly, identify the optimal experimental strategy from those available.

**Fig 5:**
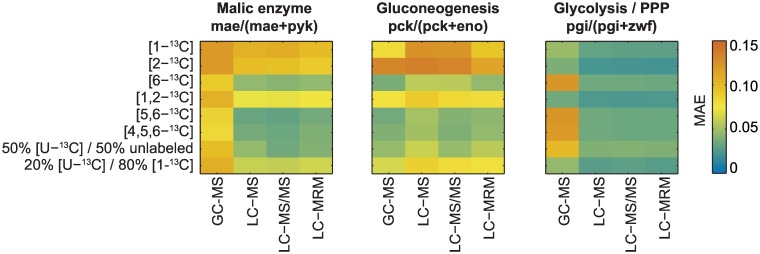
Optimizing experimental design to improve the estimation of three flux ratios in *Bacillus subtilis* central carbon metabolism. Mean absolute errors on the test dataset of three flux ratio predictors applied to *in silico* data simulated with different experimental setups. GC-MS—gas chromatography mass spectrometry, LC-MS—liquid chromatography mass spectrometry; LC-MS/MS—liquid chromatography-tandem mass spectrometry; LC-MRM—liquid chromatography with multiple reaction monitoring information; MAE—mean absolute error; PPP—pentose phosphate pathway.

We demonstrated this feature of SUMOFLUX by testing different settings for the *B*. *subtilis* labeling experiment. Using the same reference flux dataset as above, we simulated the ^13^C metabolite labeling patterns for eight different glucose-labeling strategies. For each label, we simulated the measurements that could be obtained using four different measurement techniques: GC-MS analyses of amino acids, liquid chromatography LC–MS of intact intracellular metabolites, LC-MS/MS analyses of intact metabolites and their fragments [28] (S7 Table), and all individual MS/MS traces used in multiple reaction monitoring of metabolites (S8 Table). For each of the 32 experimental setups, we rapidly trained random forest predictors for the malic enzyme, gluconeogenesis, and glycolysis/PPP flux ratios, and assessed their performance *in silico* on the test dataset. To compensate for the different number of features, and avoid over-fitting, we introduced a feature selection procedure using cross-validation on the training dataset prior to training (see Materials and Methods for details). As expected, flux calculability depends on the flux ratio of interest, the tracer, and the measurement platform (Fig 5). For the malic enzyme and glycolysis ratios, LC-based methods are preferable to GC-MS. Tracers such as [6-^13^C], [5,6-^13^C], or [4,5,6-^13^C] glucose offer the best overall accuracy (MAE < 0.05). Any of these tracers could be selected to quantify the three flux ratios in a single experiment. For specific flux ratios, the average error was reduced to about MAE 0.02–0.03 by selecting specific tracers. However, when taking into account the cost of tracers, a labeling experiment using 50% [U-^13^C] and 50% naturally labeled glucose might be seen as a compromise between prediction accuracy and cost. This analysis underlines the ease with which an experimental design, targeted to address specific biological questions, can be implemented using the SUMOFLUX workflow.

## Discussion

We have developed a generalized method for targeted analysis of ^13^C metabolic flux ratios, that builds on surrogate modeling (SUMOFLUX), i.e. uses a synthetic dataset to train a machine learning predictor to estimate a given flux ratio directly from ^13^C-data. Synthetic datasets are constructed *in silico* on the sole basis of four easily accessible inputs: a stoichiometric model of metabolism, a list of possible metabolic substrates and their byproducts, a configuration of the ^13^C-substrate, and a list of measurable metabolites with measurement error. These inputs are sufficient to generate a representative synthetic dataset covering a broad range of fluxes and flux ratios. A random forest predictor is then trained on this dataset to capture the relationship between simulated ^13^C-data and the flux ratio of interest, that holds true for all of the simulated data points. Therefore, the same predictor can be used to estimate flux ratios for normal cells, as well as for knock-out mutants without the need for additional information on physiological parameters or their uptake/consumption rates. Due to the fact that the SUMOFLUX predictor targets only a single flux ratio at a time, it is very efficient in assessing calculability and eventually estimating flux values from real data. This feature is particularly relevant when tackling complex fluxes [29,30], as dozens of different experimental designs can be trialed within a few hours. If necessary, measurement data from parallel experiments using different ^13^C tracers can be combined and passed as input features into the SUMOFLUX workflow. This approach has been proven to improve flux estimates in certain cases [31,32]. The surrogate modeling of cells grown in rich media with multiple substrates is made possible because carbon labeling experiments can be simulated for large or even genome-wide networks [33], inclusive of all the key metabolic pathways. Overall, SUMOFLUX is generally applicable to virtually any combination of metabolic model (organism), medium composition, isotopic tracer, or measurement technique.

The crucial step in SUMOFLUX is the construction of the synthetic data used for predictor training. To obtain representative data, it is extremely important that the surrogate model be based on realistic assumptions of the metabolic network and experimental measurement accuracy. Prior knowledge can be integrated into the sampling procedure to limit the space of flux distributions and potentially improve the predictor’s performance. Network simplification and constraining bear some risks. The metabolic model should encode all possible metabolic reactions such that ^13^C-patterns can be correctly assigned to the underlying flux states. If a reaction is omitted from the surrogate model, the predictor will provide biased estimates. Although the omission of reactions from a model leads to better accuracy *in silico*, that step would only be justified if the reaction was proven to be inactive under all conditions tested, e.g. by biochemical assay or enzyme quantification. Unless such information is available, it is recommended that all reactions be included in the model in order to achieve robust predictor training. For similar reasons, it is equally important to provide a real-life or conservative error model of the measurement data. According to *in silico* testing, overestimating noise in the simulated dataset does not lead to overestimating predictor’s accuracy, on the contrary to underestimating noise. In our experience, a valid sanity check is to verify that the simulated data distribution covers the measured mass isotopomer fractions by comparing the distributions of simulated and experimental data. Another indicator of potential discrepancies between the simulated and experimental data is the difference between the distributions of inter-quantile ranges of the flux ratio predictions, which can be tested with a nonparametric test, such as Wilcoxon-Mann-Whitney. With these simple procedures, errors in the metabolic model, substrate composition, or experimental measurement can be detected.

Targeted flux ratio analysis using SUMOFLUX is best suited to the assessment of flux ratio to a high accuracy, on selected metabolic nodes, or when mid to large throughput is necessary. High-throughput is made possible by the speed of the approach and by the fact that only ^13^C-data are required. Once trained, the predictor can be applied to estimate flux ratios for all tested samples simultaneously. A further optimization of experimental measurement time can be explored by including feature selection during training to identify the most informative as well as negligible ^13^C-features. SUMOFLUX complements the alternative global isotopomer balancing and iterative fitting method (^13^C-MFA), which requires measurements of uptake/consumption rates, and more detailed analyses of each dataset, but provides net flux estimates for all reactions in the model. Our short comparison with the data of 8 *E*. *coli* strains demonstrated that the two approach deliver consistent flux ratio estimates. In some instances, the confidence of SUMOFLUX estimates was better. Hence, it could be used before 13C-MFA to increase its performance. In this case, multiple flux ratios could be estimated independently to obtain experimental information on different degrees of freedom prior to applying global ^13^C flux analysis methods [34].

In principle, the concept of SUMOFLUX can be extended to isotopically non-stationary data. The simulation of dynamic ^13^C-data can be completed with the inclusion of metabolite concentrations in the sampling procedure with simulation of ^13^C dynamics at predefined time points to be matched in the experiment. The training of flux predictors from isotopically non-stationary data can use the same procedure outlined for stationary data, even though it is substantially more demanding because of the requirement to sample an increased number of degrees of freedom and measurable labeling features. However, it must be stressed that non-stationary labeling experiments are much more labor-intensive and data demanding, and can be performed only at low throughput [35]. For practical reasons, the traditional approach of flux estimation by both global [35,36] or local [37] iterative fitting is better suited to the analysis of small-scale non-stationary labeling experiments.

Overall, the concepts underlying the proposed SUMOFLUX workflow are easily transferrable and can be applied alone, or in combination with other methods, to address different flux analyses questions. We believe that SUMOFLUX has the potential to become a core tool in the analysis of metabolic fluxes, and opens new possibilities for high-throughput flux profiling of a wide variety of metabolic systems.

## Materials and methods

### Network construction

Metabolic network with carbon atom transitions and the lists of input and output metabolites are defined by the user and are represented in the mat-file format required by the INCA software [9]. In order to reduce the dependency on the biomass vector coefficients, a separate output flux is defined for each of the biomass precursors, therefore biomass precursors are also added to the list of outputs. The substrates are defined as unbalanced compounds and do not participate in the stoichiometric equation system.

### Flux sampling and ratio calculation

In the flux sampling procedure, the definitions of net, exchange, forward and backward fluxes are used [38]. By default, the lower and upper bounds for reversible reactions are set to [-100 100], for irreversible reactions to [0 100], and the major uptake flux is set to 10. First, the initial net flux solution is found by minimizing the sum of squared fluxes with stoichiometric constraints, inequality constraints on the output fluxes, and flux bounds using the MATLAB solver fmincon. Second, a cohort of net flux vectors is generated with Monte Carlo sampling by adding linear combinations of null vectors of the stoichiometric matrix with random coefficients to the initial flux solution. Third, for each net flux, an exchange flux value is randomly generated in the order of magnitude relative to the net flux defined by the user (by default 1), and forward and backward flux values are calculated accordingly. Optionally, to achieve uniform coverage of values for a particular flux ratio or set of ratios, the ratio range is split into segments (for example, [0 0.1], [0.1 0.2]… [0.9 1]), and the flux sampling procedure is repeated for each segment with the end points set as flux ratio constraints in the first step. The flux ratio of interest is calculated for each of the flux vectors with a formula defined by the user.

### ^13^C labeling patterns simulation and measured data simulation

Given the label of the substrate(s) and the list of metabolites and fragments, metabolite labeling patterns are simulated for each flux solution using the INCA software [9]. The INCA ‘simulate’ procedure is integrated into the SUMOFLUX workflow and is called internally for each of the sampled flux vectors. In case parallel computing is available, this procedure is parallelized.

The measurement data is simulated by extracting the measured compounds from the simulated data matrix and adding uniform noise to the measurements (0.01 by default). After adding noise, the mass distribution vectors for each metabolite are normalized.

### Prediction procedures

#### Preparing the training and test samples and building the predictor

For each prediction task, a subset of data points is extracted from the entire simulated dataset to ensure that the dependent variable (flux ratio of interest) is uniformly distributed on the feasible range. The flux ratio values of the whole dataset are binned into segments (for example, [0 0.1], [0.1 0.2] … [0.9 1]), and from each bin an equal amount of samples is drawn without replacement. This simulated subset is randomly divided in proportion 2:1 to form the training and test subsets. The MATLAB randomforest package (https://code.google.com/archive/p/randomforest-matlab/) modified to perform quantile regression [21] is used to build the predictor. The regRF_train function is called with ntree trees and mtry variables for the node split (by default, ntree = 100 and mtry = 20) to train the predictor on the training dataset. The predictor’s estimates on the test dataset are obtained with the function regRF_predict, and the performance is assessed by calculating the mean absolute error between the estimates and the known simulated flux ratio values.

#### Cross-validation procedure

The five-fold cross-validation procedure is performed in the following way: the training dataset is randomly divided into five parts of equal size. One of the parts is the validation subset and is used to assess the predictor performance, whereas the other four are used to train the predictor with a certain set of parameters. The procedure is repeated until all subsets were used as validation subsets once, and the error is averaged across all the subsets.

#### Prediction quantile calculation

The prediction quantile calculation is based on the quantile regression forest algorithm [21]. To calculate prediction quantiles for a new data point, regRF_predict function is called with extra_options parameters (extra_options.predict_all = true, extra_options.nodes = 1). For each tree in the forest, the terminal node where the new data point propagated is recorded. The same procedure is repeated for all data points in the training dataset. For each data point in the training dataset, a weight is calculated based on how many times this data point and the new data point propagated to the same terminal node. The data points in the training dataset are sorted by the value of the dependent variable (flux ratio of interest), and the weights cumulative function is calculated. This function is used to estimate the prediction median (50%-quantile) and to report the [10% 90%] prediction interval.

#### Feature selection

If the number of features (measured labeling patterns) is large, the predictor’s performance on the test dataset might decrease due to overfitting to the training dataset. To reduce the chance of overfitting, feature selection is performed using the cross-validation procedure. First, the predictor is trained based on all available features, and its performance is assessed with cross-validation. Second, the features are ranked according to the feature importance value calculated during training by regRF_train function. Third, the predictor is trained based on [50%, 25%, 10%, 5%] of the most important features. The mean absolute error is calculated with cross-validation. The percentage of features with the smallest MAE is selected for the further training.

#### Noise and exchange flux magnitude sensitivity analysis

To assess the sensitivity of the flux ratio predictors to noise and exchange flux magnitude, a set of 60’000 net fluxes was sampled, and the exchange flux values were randomly generated using each of the tested parameters as the upper bound: [0.1 1 10 100]. Isotope labeling was simulated for each of the four flux datasets as described previously. For each dataset, random noise was added using each of the tested parameters as the upper bound: [0 0.01 0.05 0.1]. The same separation into training and test subsets was used for all 16 simulated datasets. The training and testing was performed on the subsets with matching parameters. To assess the effect of the mismatched training and testing parameters, datasets with fixed noise level (0.01) and four different exchange flux magnitude values, or datasets with fixed exchange flux magnitude (1) and four different noise levels were used. The inter-quantile range distributions of the testing datasets were compared using right-tailed Wilcoxon-Mann-Whitney rank sum test (ranksum function in MATLAB).

#### ^13^C Metabolic flux analysis (MFA)

^13^C-MFA was performed with INCA software [9] in MATLAB 2013a (MathWorks Inc). The measured labeling data and glucose uptake rates [15] were used as inputs to the model. Three sets of data were used to constrain the model: the data from [1-^13^C] experiment only, the data from [U-^13^C] experiment only, and the combined dataset from both experiments. Each experimental strain was analyzed separately. Best fit flux solution and 95% flux confidence intervals were calculated with the parameter continuation procedure (‘continuate’). The [10% 90%] quantiles of the flux ratio distributions were estimated from the 1000 solutions found with the optimization procedure (‘estimate’) for each experimental strain.

### Experimental data

Experimental data for *E*. *coli* and *B*. *subtilis* central carbon metabolism studies were downloaded from the supplementary materials available for the corresponding papers [15,16].

### Code availability

MATLAB code for SUMOFLUX and example scripts are available at http://www.imsb.ethz.ch/research/zamboni/resources.html All scripts are compatible with MATLAB 2013a (MathWorks Inc).

## Supporting Information

S1 Fig *Escherichia coli* and *Bacillus subtilis* metabolic networks used in the study. (a) *E*. *coli* metabolic network used for the simulations. (b) *B*. *subtilis* metabolic network used for the simulations. Biomass precursor fluxes are depicted with a dashed arrow.
(TIF)

S2 Fig Choosing the SUMOFLUX predictor’s parameters and sample size. (a) SUMOFLUX performance assessment with 5-fold cross-validation (CV) with different values of ntree and mtry parameters. (b) SUMOFLUX performance on the test dataset for different sample sizes.
(TIF)

S3 Fig Comparison of SUMOFLUX and analytic formulas for key flux ratio estimation in *B*. *subtilis* central metabolism. From left to right: a schematic representation of the flux ratio; density plot representing SUMOFLUX estimates versus the true flux ratios for *in silico* data; comparison of the SUMOFLUX and analytic formula estimates for the experimental data; density plot representing analytic formula estimates versus the true flux ratios for *in silico* data. Vertical error bars in the third panel represent [10% 90%] SUMOFLUX prediction quantiles, horizontal error bars represent standard deviation of the analytic formula estimate. (a) Glycolysis versus PPP. (b) PEP fraction from gluconeogenesis. (c) Pyruvate fraction from the malic enzyme flux. (d) Oxaloacetate fraction from anaplerosis from pyruvate. Ratios were estimated for the experiment with 80% [1-^13^C] and 20% [U-^13^C] glucose. 6PG– 6-phosho-D-gluconate; αKG– α-ketoglutarate; AcCoA—acetyl-CoA; F6P –fructose-6-phosphate; Fum—fumarate; G6P –glucose-6-phosphate; ICT—isocitrate; MAE—mean absolute error; Mal—malate; PCC—Pearson correlation coefficient; PEP—phosphoenolpyruvate; PGA—phosphoglycerate; PPP—pentose phosphate pathway.
(TIF)

S4 Fig Comparison of SUMOFLUX and ^13^C-MFA analysis of *E*. *coli* central metabolism. Comparison of the SUMOFLUX and ^13^C-MFA flux ratio estimates for the experimental data. Error bars represent [10% 90%] prediction quantiles. (a) ^13^C-MFA flux ratios were calculated for the optimal solutions fitted to the combined data of [1-^13^C] and [U-^13^C] glucose labeling experiments. (b) ^13^C-MFA flux ratios were calculated for the optimal solutions fitted to the data of [1-^13^C] glucose labeling experiment only. (c) ^13^C-MFA flux ratios were calculated for the optimal solutions fitted to the data of [U-^13^C] glucose labeling experiment only. E-D—Entner-Doudoroff pathway; PCC—Pearson correlation coefficient; PPP—pentose phosphate pathway.
(TIF)

S5 Fig Robustness analysis of analytic formulas in terms of noise and exchange flux magnitude. Mean absolute errors on the test dataset of five analytic formulas applied to *in silico* data with different amount of measurement noise and exchange flux magnitude. The dashed rectangle indicates the normal range of noise (0.01) and exchange flux magnitude (10 times the net flux). E-D—Entner-Doudoroff pathway, MAE—mean absolute error; PPP—pentose phosphate pathway.
(TIF)

S6 Fig Distributions of inter-quantile ranges of the flux ratio predictions might indicate incompatibility of training and testing datasets. (a) Histograms representing the [10% 90%] inter-quantile ranges of the pyruvate fraction from the malic enzyme flux ratio predictions on the test dataset. The noise level in the training dataset is varied along the y-axis, the noise level in the testing dataset is varied along the x-axis. The exchange flux magnitude was set to 1. The histograms in black represent cases of compatible training and testing datasets with the same assumptions on the noise level and exchange flux magnitude. (b) Histograms representing the [10% 90%] inter-quantile ranges of the pyruvate fraction from the malic enzyme flux ratio predictions on the test dataset. The exchange flux magnitude in the training dataset is varied along the y-axis, the exchange flux magnitude in the testing dataset is varied along the x-axis. The noise level was set to 0.01. The histograms in black represent cases of compatible training and testing datasets with the same assumptions on the noise level and exchange flux magnitude. WMW—p-value of the Wilcoxon-Mann-Whitney right tail test comparing the distributions of inter-quantile range of each testing dataset to the distribution of inter-quantile range of the testing dataset compatible with the noise and exchange level assumptions of the training dataset (diagonal plots in black). Low WMW values indicate that the median of the inter-quantile range distribution of the corresponding testing dataset is significantly larger than the median of the inter-quantile range distribution of the testing dataset compatible with the assumptions of the training dataset, which indicates that these assumptions are incompatible with the current testing set.
(TIF)

S7 Fig SUMOFLUX estimates for the relative contributions of fluxes to the oxaloacetate pool for the published *E*. *coli* data. (a) A schematic representation of the glyoxylate shunt, TCA cycle and anaplerosis from PEP fluxes contributing to the formation of oxaloacetate. (b) SUMOFLUX prediction of the relative contributions of the anaplerotic flux from phosphoenolpyruvate, glyoxylate shunt and TCA cycle flux to the oxaloacetate pool for the *in silico* test dataset. The error bars represent [10% 90%] prediction quantiles. Data from experiment with 20% [U-^13^C] and 80% naturally labeled glucose. αKG– α-ketoglutarate; AcCoA—acetyl-CoA; Fum—fumarate; Gox—glyoxylate; ICT—isocitrate; Mal—malate; PEP—phosphoenolpyruvate; TCA cycle—tricarboxylic acid cycle.
(TIF)

S1 Table *E*. *coli* network of central carbon metabolism used throughout the study. (DOCX)

S2 Table Metabolites and metabolite fractions inferable from amino-acid measurements with GC-MS used in *E*. *coli* and *B*. *subtilis* studies. The metabolites and metabolite fragments were simulated in the *in silico* dataset for *E*. *coli* and *B*. *subtilis*.
(DOCX)

S3 Table Analytic formulas used to calculate flux ratios in central carbon metabolism of *E*. *coli* and *B*. *subtilis*. (DOCX)

S4 Table SUMOFLUX predictions and analytic formulas’ estimates of five flux ratios in central metabolism of *E*. *coli* for eight strains grown on 100% [1-^13^C] or 20% [U-^13^C] and 80% naturally labeled glucose; and for 121 *B*. *subtilis* strains grown on combination of 80% [1-13C] and 20% [U-13C] glucose. Flux ratio estimates (median) and 10% and 90% prediction quantiles are reported.
(XLSX)

S5 Table ^13^C-MFA analysis of *E*. *coli* for eight strains grown on 100% [1-^13^C] or 20% [U-^13^C] and 80% naturally labeled glucose. The best fit flux values and 95% confidence intervals for the fluxes estimated using parameter continuation procedure in INCA software. The prediction quantiles for the flux ratios were calculated from the flux ratio distributions of 1000 flux solutions found with the optimization procedure.
(XLSX)

S6 Table SUMOFLUX predictions for metabolic origin of oxaloacetate for eight *E*. *coli* strains grown on 20% [U-^13^C] and 80% naturally labeled glucose. Flux ratio estimates (median) and 10% and 90% prediction quantiles are reported.
(DOCX)

S7 Table Metabolites and metabolite fractions measurable with LC-MS or LC-MS/MS methods. The metabolites and fragments that were used for *in silico* experimental design.
(DOCX)

S8 Table MRM transitions of the metabolites and metabolite fractions measurable with LC-MS/MS methods. (XLSX)
