# Supplementary figures and images for: SUMOFLUX: A Generalized Method for Targeted 13C Metabolic Flux Ratio Analysis
Source: PLoS Comput Biol. 2016 Sep 14;12(9):e1005109. doi: 10.1371/journal.pcbi.1005109 (PMC5023139; doi:10.1371/journal.pcbi.1005109)

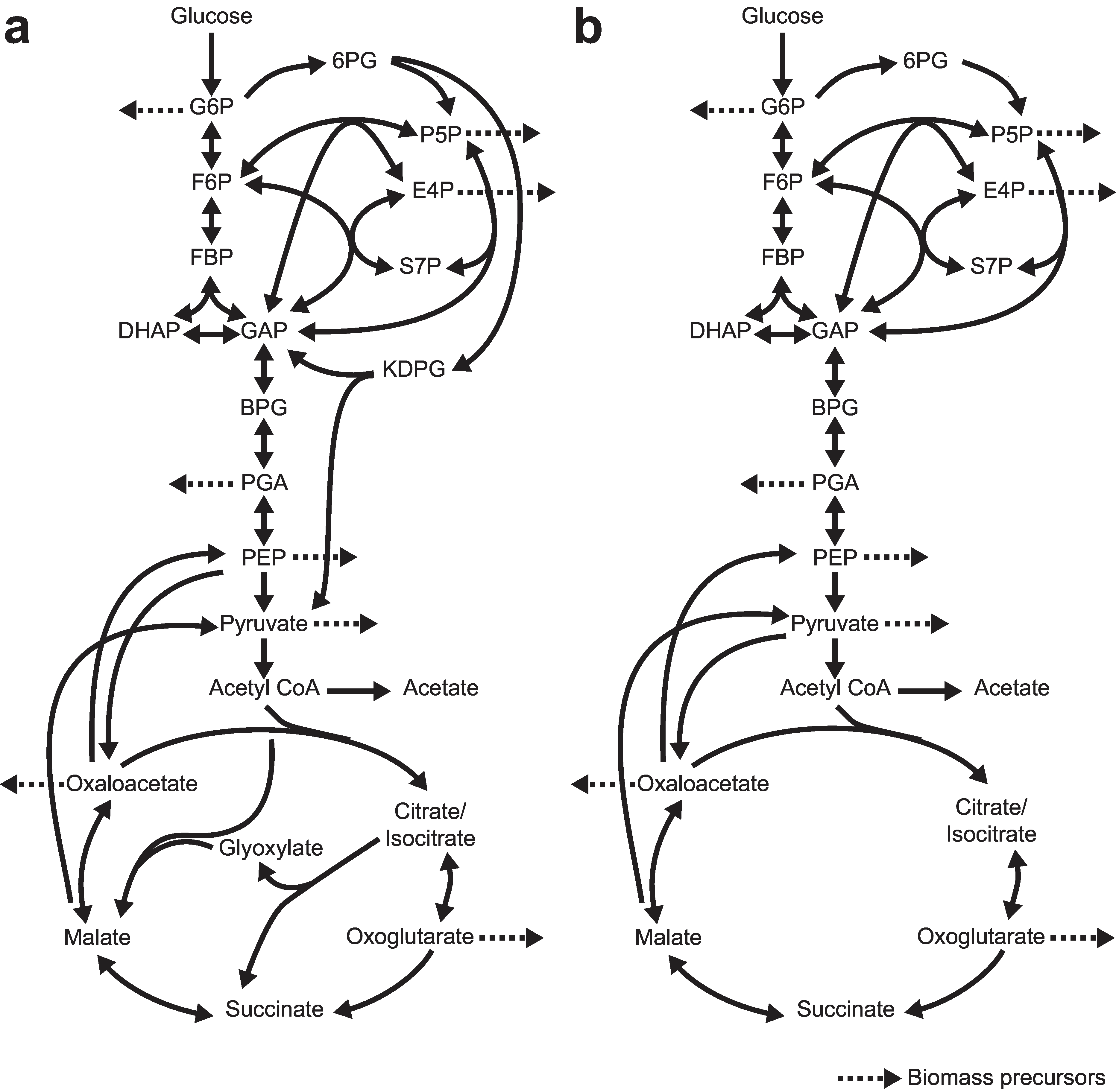

Supplement: S1 Fig — (a) E. coli metabolic network used for the simulations. (b) B. subtilis metabolic network used for the simulations. Biomass precursor fluxes are depicted with a dashed arrow. (TIF) [file pcbi.1005109.s001.tif]

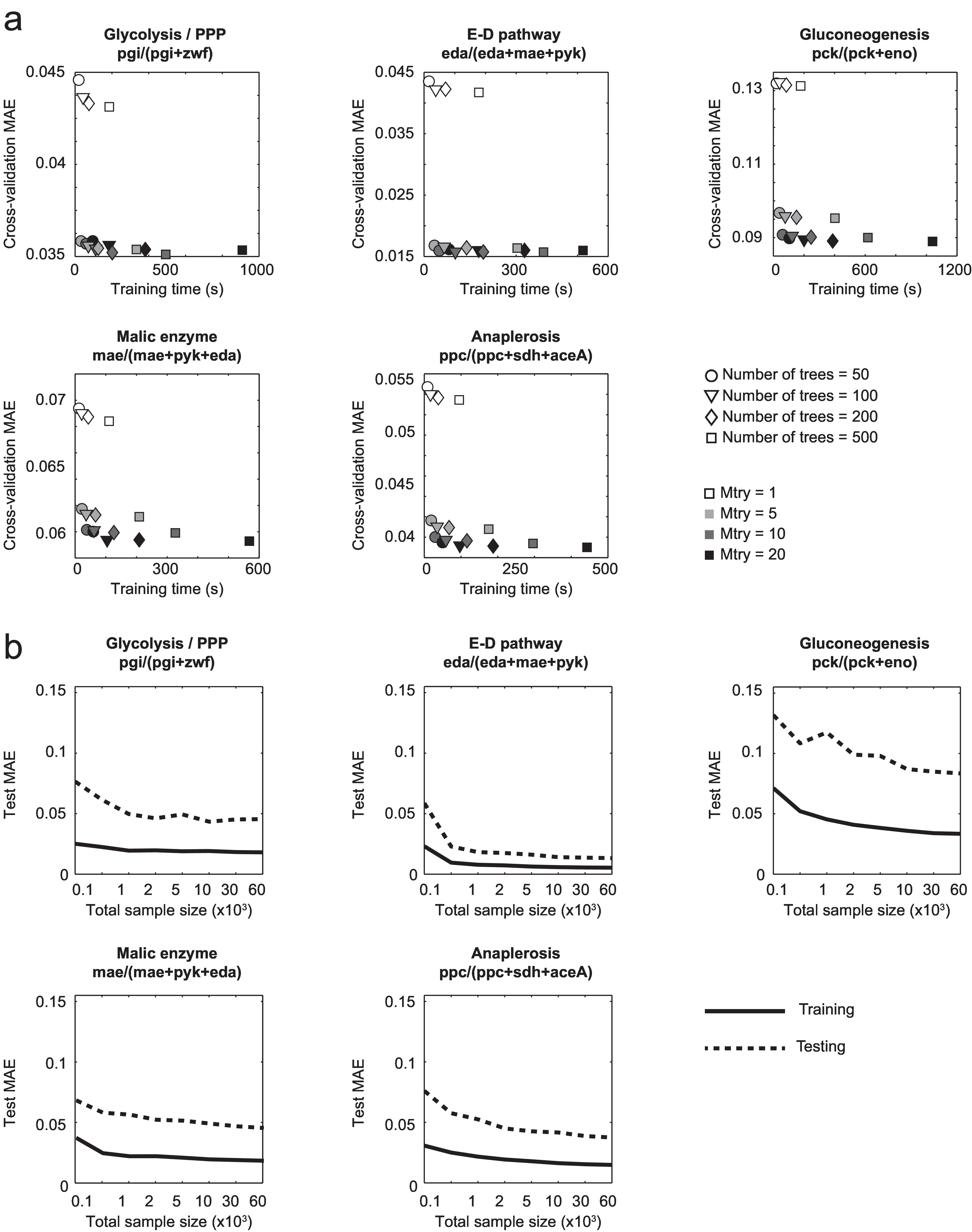

Supplement: S2 Fig — (a) SUMOFLUX performance assessment with 5-fold cross-validation (CV) with different values of ntree and mtry parameters. (b) SUMOFLUX performance on the test dataset for different sample sizes. (TIF) [file pcbi.1005109.s002.tif]

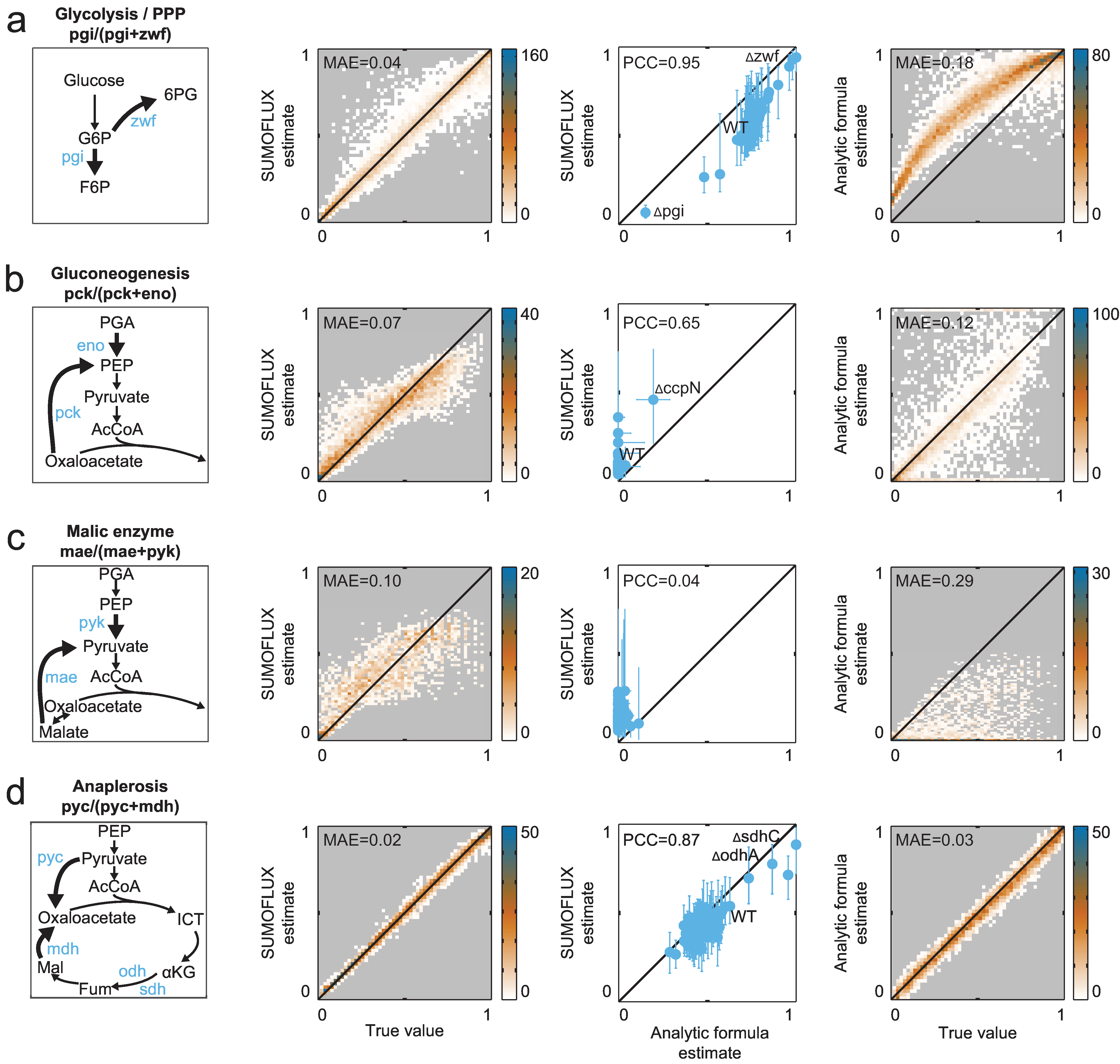

Supplement: S3 Fig — From left to right: a schematic representation of the flux ratio; density plot representing SUMOFLUX estimates versus the true flux ratios for in silico data; comparison of the SUMOFLUX and analytic formula estimates for the experimental data; density plot representing analytic formula estimates versus the true flux ratios for in silico data. Vertical error bars in the third panel represent [10% 90%] SUMOFLUX prediction quantiles, horizontal error bars represent standard deviation of the analytic formula estimate. (a) Glycolysis versus PPP. (b) PEP fraction from gluconeogenesis. (c) Pyruvate fraction from the malic enzyme flux. (d) Oxaloacetate fraction from anaplerosis from pyruvate. Ratios were estimated for the experiment with 80% [1-13C] and 20% [U-13C] glucose. 6PG– 6-phosho-D-gluconate; αKG– α-ketoglutarate; AcCoA—acetyl-CoA; F6P –fructose-6-phosphate; Fum—fumarate; G6P –glucose-6-phosphate; ICT—isocitrate; MAE—mean absolute error; Mal—malate; PCC—Pearson correlation coefficient; PEP—phosphoenolpyruvate; PGA—phosphoglycerate; PPP—pentose phosphate pathway. (TIF) [file pcbi.1005109.s003.tif]

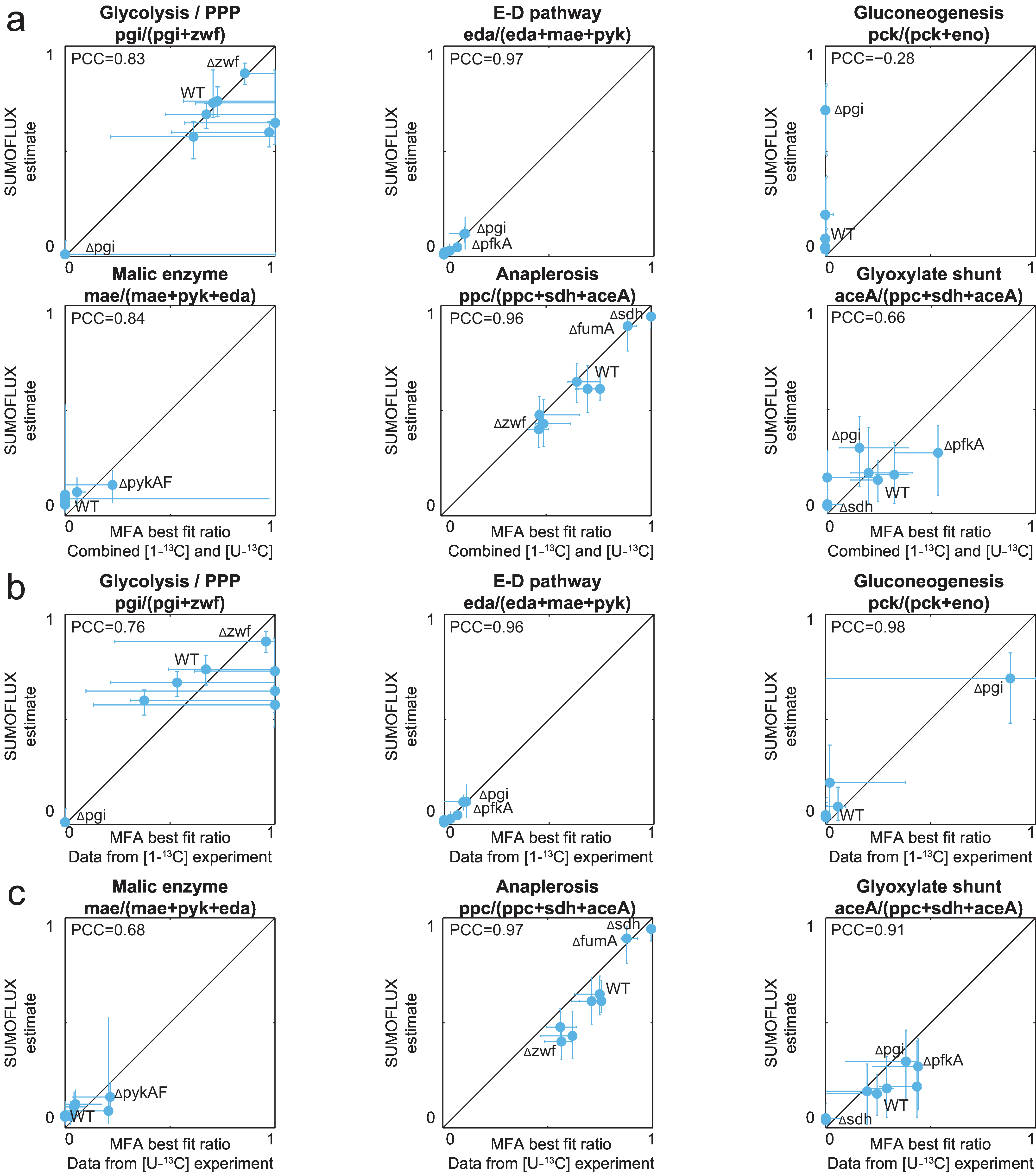

Supplement: S4 Fig — Comparison of the SUMOFLUX and 13C-MFA flux ratio estimates for the experimental data. Error bars represent [10% 90%] prediction quantiles. (a) 13C-MFA flux ratios were calculated for the optimal solutions fitted to the combined data of [1-13C] and [U-13C] glucose labeling experiments. (b) 13C-MFA flux ratios were calculated for the optimal solutions fitted to the data of [1-13C] glucose labeling experiment only. (c) 13C-MFA flux ratios were calculated for the optimal solutions fitted to the data of [U-13C] glucose labeling experiment only. E-D—Entner-Doudoroff pathway; PCC—Pearson correlation coefficient; PPP—pentose phosphate pathway. (TIF) [file pcbi.1005109.s004.tif]

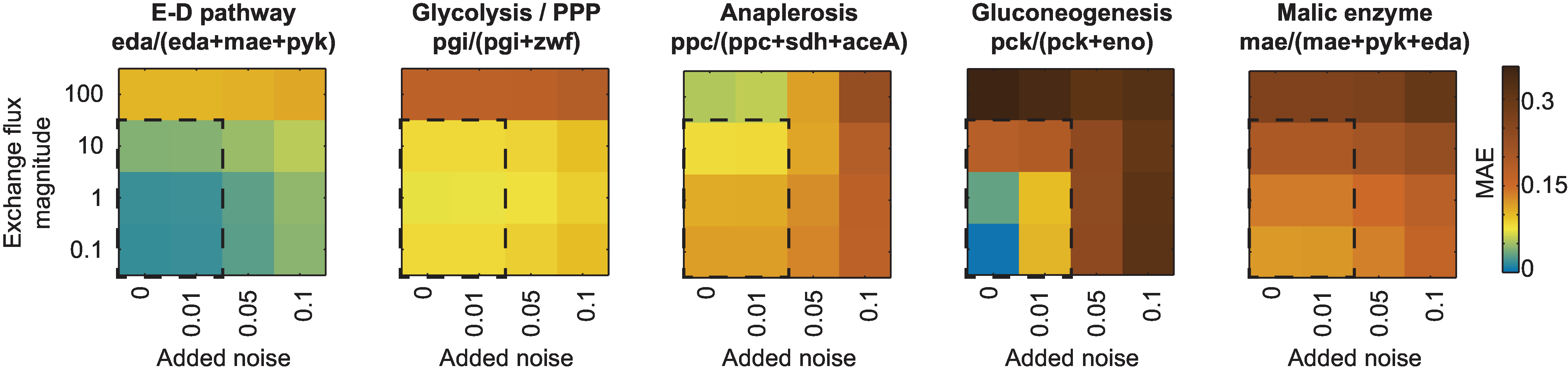

Supplement: S5 Fig — Mean absolute errors on the test dataset of five analytic formulas applied to in silico data with different amount of measurement noise and exchange flux magnitude. The dashed rectangle indicates the normal range of noise (0.01) and exchange flux magnitude (10 times the net flux). E-D—Entner-Doudoroff pathway, MAE—mean absolute error; PPP—pentose phosphate pathway. (TIF) [file pcbi.1005109.s005.tif]

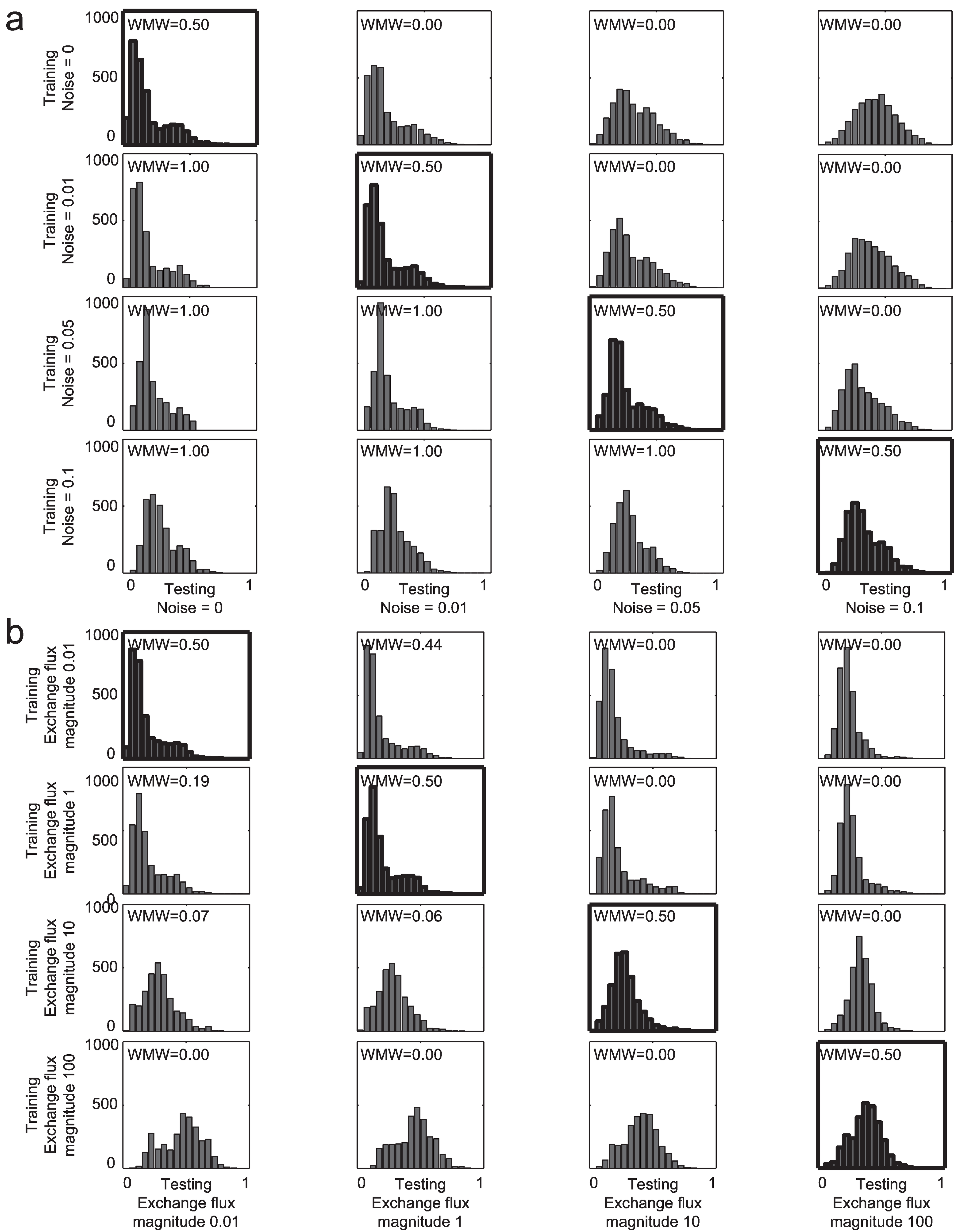

Supplement: S6 Fig — (a) Histograms representing the [10% 90%] inter-quantile ranges of the pyruvate fraction from the malic enzyme flux ratio predictions on the test dataset. The noise level in the training dataset is varied along the y-axis, the noise level in the testing dataset is varied along the x-axis. The exchange flux magnitude was set to 1. The histograms in black represent cases of compatible training and testing datasets with the same assumptions on the noise level and exchange flux magnitude. (b) Histograms representing the [10% 90%] inter-quantile ranges of the pyruvate fraction from the malic enzyme flux ratio predictions on the test dataset. The exchange flux magnitude in the training dataset is varied along the y-axis, the exchange flux magnitude in the testing dataset is varied along the x-axis. The noise level was set to 0.01. The histograms in black represent cases of compatible training and testing datasets with the same assumptions on the noise level and exchange flux magnitude. WMW—p-value of the Wilcoxon-Mann-Whitney right tail test comparing the distributions of inter-quantile range of each testing dataset to the distribution of inter-quantile range of the testing dataset compatible with the noise and exchange level assumptions of the training dataset (diagonal plots in black). Low WMW values indicate that the median of the inter-quantile range distribution of the corresponding testing dataset is significantly larger than the median of the inter-quantile range distribution of the testing dataset compatible with the assumptions of the training dataset, which indicates that these assumptions are incompatible with the current testing set. (TIF) [file pcbi.1005109.s006.tif]

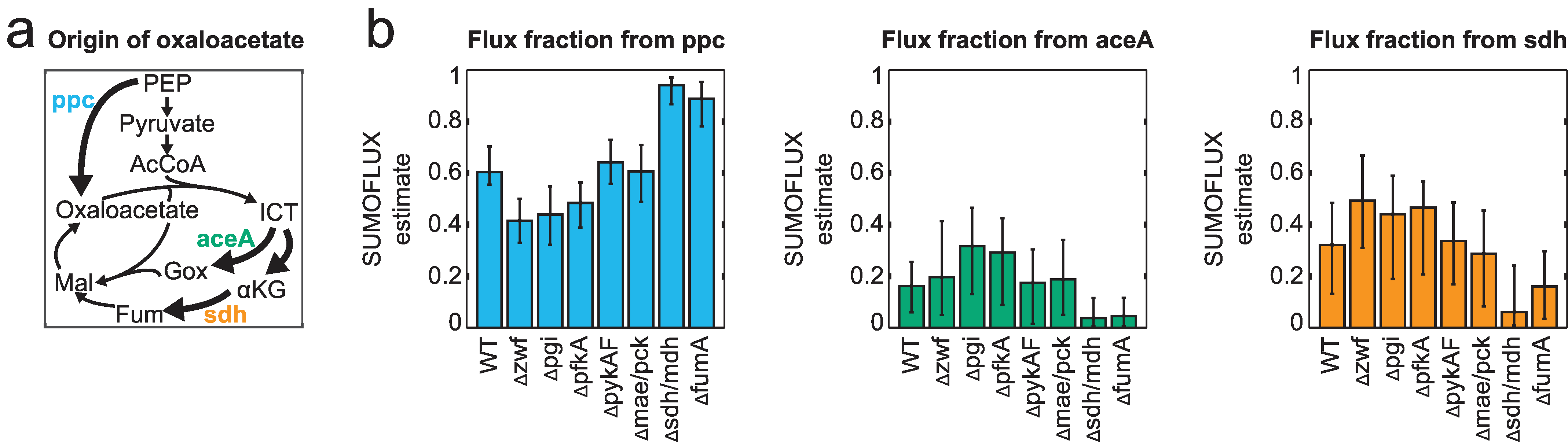

Supplement: S7 Fig — (a) A schematic representation of the glyoxylate shunt, TCA cycle and anaplerosis from PEP fluxes contributing to the formation of oxaloacetate. (b) SUMOFLUX prediction of the relative contributions of the anaplerotic flux from phosphoenolpyruvate, glyoxylate shunt and TCA cycle flux to the oxaloacetate pool for the in silico test dataset. The error bars represent [10% 90%] prediction quantiles. Data from experiment with 20% [U-13C] and 80% naturally labeled glucose. αKG– α-ketoglutarate; AcCoA—acetyl-CoA; Fum—fumarate; Gox—glyoxylate; ICT—isocitrate; Mal—malate; PEP—phosphoenolpyruvate; TCA cycle—tricarboxylic acid cycle. (TIF) [file pcbi.1005109.s007.tif]
