# Supplementary material for: SUMOFLUX: A Generalized Method for Targeted 13C Metabolic Flux Ratio Analysis
Source: PLoS Comput Biol. 2016 Sep 14;12(9):e1005109. doi: 10.1371/journal.pcbi.1005109 (PMC5023139; doi:10.1371/journal.pcbi.1005109)
Supplement: S1 Table — (DOCX) [file pcbi.1005109.s008.docx]

**S1 Table. *E. coli* network of central carbon metabolism used throughout the study.**

| Reaction type | Reaction | Stoichiometry and carbon transitions |
| --- | --- | --- |
| Uptake  and secretion | glc_up | glucose (abcdef) -> G6P (abcdef) |
|  | CO2up | CO2in (a) -> CO2 (a) |
|  | accoa_ac | AcCoA (ab) -> Ac (ab) |
|  | ac_out | Ac (ab) -> Acetate (ab) |
|  | co2_out | CO2 (a) -> CO2out (a) |
| Biomass  precursors | G6P_bm | G6P (abcdef) -> G6Pbm (abcdef) |
|  | PGA_bm | PGA (abc) -> PGAbm (abc) |
|  | P5P_bm | P5P (abcde) -> P5Pbm (abcde) |
|  | PEP_bm | PEP (abc) -> PEPbm (abc) |
|  | PYR_bm | PYR (abc) -> PYRbm (abc) |
|  | OGA_bm | OGA (abcde) -> OGAbm (abcde) |
|  | OAA_bm | OAA (abcd) -> OAAbm (abcd) |
|  | E4P_bm | E4P (abcd) -> E4Pbm (abcd) |
| Glycolysis | pgi | G6P (abcdef) <-> F6P (abcdef) |
|  | pfk | F6P (abcdef) -> FBP (abcdef) |
|  | fba | FBP (abcdef) -> DHAP (cba) + GAP (def) |
|  | tpi | DHAP (abc) <-> GAP (abc) |
|  | gapdh | GAP (abc) -> BPG (abc) |
|  | bpg | BPG (abc) -> PGA (abc) |
|  | eno | PGA (abc) -> PEP (abc) |
|  | pyk | PEP (abc) -> PYR (abc) |
| Pentose  Phosphate  Pathway | zwf | G6P (abcdef) -> PG6 (abcdef) |
|  | gnd | PG6 (abcdef) -> P5P (bcdef) + CO2 (a) |
|  | TK1 | P5P (abcde) + P5P (fghij) <-> GAP (cde) + S7P (abfghij) |
|  | TK2 | P5P (abcde) + E4P (fghi) <-> GAP (cde) + F6P (abfghi) |
|  | TA | S7P (abcdefg) + GAP (hij) <-> E4P (defg) + F6P (abchij) |
| Entner-Doudoroff | edp1 | PG6 (abcdef) -> KDPG (abcdef) |
|  | edp2 | KDPG (abcdef) -> PYR (abc) + GAP (def) |
| TCA cycle | pdh | PYR (abc) -> AcCoA (bc) + CO2 (a) |
|  | citl | OAA (cdef) + AcCoA (ab) -> Cit (fedcba) |
|  | idh | Cit (abcdef) <-> OGA (abcef) + CO2 (d) |
|  | sdh | OGA (abcde) -> Suc (bcde) + CO2 (a) |
|  | fum | Suc (abcd) <-> Mal (abcd) |
|  | mdh | Mal (abcd) <-> OAA (abcd) |
| Glyoxylate shunt | gs1 | Cit (abcdef) -> Glx (ab) + Suc (dcef) |
|  | gs2 | Glx (ab) + AcCoA (cd) -> Mal (abcd) |
| Anaplerosis and gluconeogenesis | mae | Mal (abcd) -> PYR (abc) + CO2 (d) |
|  | pyc | PEP (abc) + CO2 (d) -> OAA (abcd) |
|  | pck | OAA (abcd) -> PEP (abc) + CO2 (d) |
