# Supplementary material for: SUMOFLUX: A Generalized Method for Targeted 13C Metabolic Flux Ratio Analysis
Source: PLoS Comput Biol. 2016 Sep 14;12(9):e1005109. doi: 10.1371/journal.pcbi.1005109 (PMC5023139; doi:10.1371/journal.pcbi.1005109)
Supplement: S2 Table — The metabolites and metabolite fragments were simulated in the in silico dataset for E. coli and B. subtilis. (DOCX) [file pcbi.1005109.s009.docx]

**S2 Table. Metabolites and metabolite fractions inferable from amino-acid measurements with GC-MS.** The following metabolites and metabolite fragments were simulated in the *in silico* dataset for *E. coli* and *B. subtilis.*

| **Metabolite** | **Abbreviation** | **Fragment carbon positions** |
| --- | --- | --- |
| Oxoglutarate | OGA15 | 1 2 3 4 5 |
| Oxoglutarate | OGA25 | 2 3 4 5 |
| Oxoglutarate | OGA12 | 1 2 |
| Phosphoenolpyruvate | PEP13 | 1 2 3 |
| Phosphoenolpyruvate | PEP23 | 2 3 |
| Phosphoenolpyruvate | PEP12 | 1 2 |
| Erythrose 4-phosphate | E4P14 | 1 2 3 4 |
| Biphosphoglycerate | BPG13 | 1 2 3 |
| Biphosphoglycerate | BPG23 | 2 3 |
| Biphosphoglycerate | BPG12 | 1 2 |
| Pyruvate | PYR13 | 1 2 3 |
| Pyruvate | PYR23 | 2 3 |
| Pyruvate | PYR12 | 1 2 |
| Oxaloacetate | OAA14 | 1 2 3 4 |
| Oxaloacetate | OAA24 | 2 3 4 |
| Oxaloacetate | OAA12 | 1 2 |
| Acetyl-CoA | AcCoA12 | 1 2 |
| Acetyl-CoA | AcCoA2 | 2 |
| Ribose-5-phosphate | P5P15 | 1 2 3 4 5 |
| Ribose-5-phosphate | P5P25 | 2 3 4 5 |
| Ribose-5-phosphate | P5P12 | 1 2 |
