# Supplementary material for: SUMOFLUX: A Generalized Method for Targeted 13C Metabolic Flux Ratio Analysis
Source: PLoS Comput Biol. 2016 Sep 14;12(9):e1005109. doi: 10.1371/journal.pcbi.1005109 (PMC5023139; doi:10.1371/journal.pcbi.1005109)
Supplement: S3 Table — (DOCX) [file pcbi.1005109.s010.docx]

**S3 Table. Analytic formulas used to calculate flux ratios in central carbon metabolism for *E. coli* and *B. subtilis* (adapted from Fischer & Sauer 2003).**

| Flux ratio | Analytic formula for *E. coli* | Analytic formula for *B. subtilis* |
| --- | --- | --- |
| Glycolysis / Pentose-phosphate pathway | $F=\frac{{BPG}_{13}-N_{13}}{0.5L_{13}\times0.5N_{13}-N_{13}}$ | $F=\frac{{BPG}_{13}-N_{13}}{0.5L_{13}\times0.5M_{13}-M_{13}}$ |
| Pyruvate from Entner-Doudoroff pathway | $F=\frac{PYR_{13}-{BPG}_{13}}{L_{13}-{BPG}_{13}}$ | n/a |
| Oxaloacetate from anaplerosis | $\left[ \begin{aligned} F \\ F*CO_{2} \end{aligned} \right]=\frac{OAA_{14}-{\alpha KG}_{25}}{\left[ \begin{matrix} PEP_{13}0 & {\alpha KG}_{25} \\ 0 PEP_{13} & PEP_{13}0 \end{matrix} \right]}$ | $\left[ \begin{aligned} F \\ F*CO_{2} \end{aligned} \right]=\frac{OAA_{14}-{\alpha KG}_{25}}{\left[ \begin{matrix} {PYR}_{13}0 & {\alpha KG}_{25} \\ 0 {PYR}_{13} & {PYR}_{13}0 \end{matrix} \right]}$ |
| PEP from gluconeogenesis | $F=\frac{PEP_{12}-{BPG}_{12}}{OAA_{12}-{BPG}_{12}}$ | $F=\frac{PEP_{12}-{BPG}_{12}}{OAA_{12}-{BPG}_{12}}$ |
| Pyruvate from malate | $F=\frac{PYR_{23}-PEP_{23}}{M_{1}\times M_{1}-PEP_{23}}$ | $F=\frac{PYR_{23}-PEP_{23}}{M_{1}\times M_{1}-PEP_{23}}$ |

M_1_-one carbon molecule labeled according to the substrate labeling

M_13_-last three carbon molecule labeled according to the substrate labeling

N_13_-three carbon molecule naturally labeled (C^13^ natural abundance 1%)

L_13_- first three carbon molecule fragment of the substrate propagated through glycolysis

αKG – α-ketoglutarate

BPG – biphosphoglycerate

OAA – oxaloacetate

PEP – phosphoenolpyruvate

PYR – pyruvate

Numbers in subscript indicate metabolic fragment
