# Supplementary material for: SUMOFLUX: A Generalized Method for Targeted 13C Metabolic Flux Ratio Analysis
Source: PLoS Comput Biol. 2016 Sep 14;12(9):e1005109. doi: 10.1371/journal.pcbi.1005109 (PMC5023139; doi:10.1371/journal.pcbi.1005109)
Supplement: S6 Table — Flux ratio estimates (median) and 10% and 90% prediction quantiles are reported. (DOCX) [file pcbi.1005109.s013.docx]

**S6 Table. SUMOFLUX predictions for metabolic origin of oxaloacetate for eight *E. coli* strains grown on 20% [U‑^13^C] and 80% naturally labeled glucose.** In brackets are given 10% and 90% prediction quantiles.

| **Strain** | **Oxaloacetate from glyoxylate** | **Oxaloacetate from TCA cycle** |
| --- | --- | --- |
| **MG1655** | 0.16  [0.06 0.26] | 0.32  [0.13 0.48] |
| **Δzwf** | 0.20  [0.05 0.41] | 0.50  [0.31 0.67] |
| **Δpgi** | 0.32  [0.13 0.47] | 0.44  [0.19 0.60] |
| **ΔpfkA** | 0.30  [0.09 0.42] | 0.47  [0.21 0.57] |
| **ΔpykAF** | 0.17  [0.02 0.30] | 0.34  [0.17 0.49] |
| **Δmae/pck** | 0.19  [0.05 0.34] | 0.29  [0.08 0.46] |
| **Δsdh/mdh** | 0.04  [0.01 0.12] | 0.06  [0.01 0.24] |
| **ΔfumA** | 0.05  [0.01 0.12] | 0.16  [0.03 0.30] |
