# Supplementary material for: SUMOFLUX: A Generalized Method for Targeted 13C Metabolic Flux Ratio Analysis
Source: PLoS Comput Biol. 2016 Sep 14;12(9):e1005109. doi: 10.1371/journal.pcbi.1005109 (PMC5023139; doi:10.1371/journal.pcbi.1005109)
Supplement: S7 Table — The metabolites and fragments that were used for in silico experimental design. (DOCX) [file pcbi.1005109.s014.docx]

**S7 Table. Metabolites and metabolite fractions measurable with LC-MS or LC-MS/MS methods.** The following metabolites and fragments were used for *in silico* experimental design.

| **Metabolite** | **Abbreviation** | **Fragment carbon positions** | **LC-MS** | **LC-MS/MS** |
| --- | --- | --- | --- | --- |
| Oxoglutarate | OGA15 | 1 2 3 4 5 | + | + |
| Oxoglutarate | OGA25 | 2 3 4 5 |  | + |
| Oxoglutarate | OGA24 | 2 3 4 |  | + |
| Phosphoenolpyruvate | PEP13 | 1 2 3 | + | + |
| Phosphoenolpyruvate | PEP23 | 2 3 |  | + |
| Biphosphoglycerate | BPG13 | 1 2 3 | + | + |
| Pyruvate | PYR13 | 1 2 3 | + | + |
| Pyruvate | PYR23 | 2 3 |  | + |
| Oxaloacetate | OAA14 | 1 2 3 4 | + | + |
| Oxaloacetate | OAA24 | 2 3 4 |  | + |
| Ribose-5-phosphate | P5P15 | 1 2 3 4 5 | + | + |
| Ribose-5-phosphate | P5P35 | 3 4 5 |  | + |
| Ribose-5-phosphate | P5P45 | 4 5 |  | + |
| Citrate | Cit16 | 1 2 3 4 5 6 | + | + |
| Citrate | Cit12 | 1 2 |  | + |
| Dihydroxyacetone phosphate | DHAP13 | 1 2 3 | + | + |
| Fructose-6-phosphate | F6P16 | 1 2 3 4 5 6 | + | + |
| Fructose-6-phosphate | F6P46 | 4 5 6 |  | + |
| Fructose-6-phosphate | F6P56 | 5 6 |  | + |
| Fructose-1,6-bisphosphate | FBP16 | 1 2 3 4 5 6 | + | + |
| Glucose-6-phosphate | G6P16 | 1 2 3 4 5 6 | + | + |
| Glucose-6-phosphate | G6P36 | 3 4 5 6 |  | + |
| Glucose-6-phosphate | G6P46 | 4 5 6 |  | + |
| Glucose-6-phosphate | G6P56 | 5 6 |  | + |
| Malate | Mal14 | 1 2 3 4 | + | + |
| Glycerate-3-phosphate | PGA13 | 1 2 3 | + | + |
| Succinate | Suc14 | 1 2 3 4 | + | + |
| Succinate | Suc13 | 1 2 3 |  | + |
| Succinate | Suc24 | 2 3 4 |  | + |
